# Magnetic Supramolecular Spherical Arrays: Direct Formation of Micellar Cubic Mesophase by Lanthanide Metallomesogens with 7‐Coordination Geometry

**DOI:** 10.1002/advs.202309226

**Published:** 2024-03-13

**Authors:** Nao Komiyama, Takahiro Ohkubo, Yoshiki Maeda, Yuya Saeki, Nobuyuki Ichikuni, Hyuma Masu, Hirofumi Kanoh, Koji Ohara, Ryunosuke Takahashi, Hiroki Wadati, Hideaki Takagi, Yohei Miwa, Shoichi Kutsumizu, Keiki Kishikawa, Michinari Kohri

**Affiliations:** ^1^ Department of Applied Chemistry and Biotechnology Graduate School of Engineering Chiba University 1–33 Yayoi‐cho, Inage‐ku Chiba 263‐8522 Japan; ^2^ Center for Analytical Instrumentation Chiba University 1–33 Yayoi‐cho, Inage‐ku Chiba 263‐8522 Japan; ^3^ Department of Chemistry Graduate School of Science Chiba University 1–33 Yayoi‐cho, Inage‐ku Chiba 263‐8522 Japan; ^4^ Faculty of Materials for Energy Shimane University 1060, Nishi‐Kawatsu‐cho Matsue Shimane 690‐8504 Japan; ^5^ Diffraction and Scattering Division Japan Synchrotron Radiation Research Institute 1‐1‐1, Kouto, Sayo‐cho Sayo‐gun Hyogo 679‐5198 Japan; ^6^ Department of Material Science Graduate School of Science University of Hyogo 3‐2‐1 Kouto, Kamigori‐cho Ako‐gun Hyogo 678‐1297 Japan; ^7^ Institute of Laser Engineering Osaka University 2–6 Yamadaoka Suita Osaka 565‐0871 Japan; ^8^ Photon Factory Institute of Materials Structure Science High Energy Accelerator Research Organization 1‐1 Oho Tsukuba Ibaraki 305‐0801 Japan; ^9^ Department of Chemistry and Biomolecular Science Faculty of Engineering Gifu University 1‐1 Yanagido Gifu 501‐1193 Japan

**Keywords:** 7‐coordinate complex, holmium, metallomesogen, micellar cubic mesophase, spherical assembly

## Abstract

Here, an unprecedented phenomenon in which 7‐coordinate lanthanide metallomesogens, which align via hydrogen bonds mediated by coordinated H_2_O molecules, form micellar cubic mesophases at room temperature, creating body‐centered cubic (BCC)‐type supramolecular spherical arrays, is reported. The results of experiments and molecular dynamics simulations reveal that spherical assemblies of three complexes surrounded by an amorphous alkyl domain spontaneously align in an energetically stable orientation to form the BCC structure. This phenomenon differs greatly from the conventional self‐assembling behavior of 6‐coordinated metallomesogens, which form columnar assemblies due to strong intermolecular interactions. Since the magnetic and luminescent properties of different lanthanides vary, adding arbitrary functions to spherical arrays is possible by selecting suitable lanthanides to be used. The method developed in this study using 7‐coordinate lanthanide metallomesogens as building blocks is expected to lead to the rational development of micellar cubic mesophases.

## Graphical abstract

An unprecedented method has been developed to prepare supramolecular spherical arrays, which have magnetic properties, directly at room temperature using 7‐coordinate lanthanide metallomesogens as building blocks. Synchrotron X‐ray scattering experiments and molecular dynamics simulations have revealed the existence of aligned complexes and their motions inside spherical assemblies composed of amorphous domains.

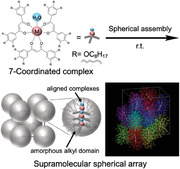

## 1 Introduction

Constructing highly ordered supramolecular structures through the self‐assembly of simple molecules is a promising approach for creating functional materials.^[^ ^1^ ^]^ In particular, 3D ordered structures consisting of spontaneously formed spherical assemblies of molecules, i.e., supramolecular spherical arrays, have recently attracted considerable attention in various fields, from drug delivery to the food industry.^[^ ^2^ ^]^ Originating from element‐scale spherical motif arrays such as metals and salts, the construction of ordered structures consisting of nanosized spherical assemblies using a variety of building blocks, such as surfactants, block copolymers, dendrons, tetrahedral molecules, and rod‐coil molecules, has been actively investigated.^[^ ^3^ ^]^ The supramolecular approach is an attractive alternative for the generation of spherical arrays. For example, a phase transition from supramolecular columns of stacked disk‐shaped molecules to supramolecular spheres has been reported.^[^ ^4^ ^]^ Although such a phase transition usually requires high temperatures, supramolecular spherical arrays such as *Im*3̅*m* (body‐centered cubic; BCC), *P*4_2_/*mnm* (Frank‐Kasper σ), and *Pm*3̅*n* (Frank‐Kasper A15) have been created.^[^ ^5^ ^]^ Examples of direct synthesis of desired supramolecular spherical arrays at room temperature are limited, and the protocol is not yet well developed.^[^ ^6^ ^]^ Furthermore, the functionalization of spherical arrays is an attractive challenge with many possibilities for separation, luminescence imaging, sensors, and actuators.^[^ ^7^ ^].^

Metallomesogens, metal complexes that exhibit mesomorphic characteristics, can form various assembled structures depending on the type of metal and the design of the ligands and thus exhibit element‐derived optical, magnetic, electrical, and redox properties.^[^ ^8^ ^]^ Control of the higher‐order structure of metallomesogens requires a balance between a rigid core moiety consisting of a metal and ligand and a flexible moiety on the ligand side chain. Metallomesogens can be designed by adapting the rules related to tuning the phase structures formed by liquid crystals based on organic molecules. Metallomesogens with rod‐shaped rigid cores and a linear coordination geometry tend to form nematic or smectic mesophases.^[^ ^9^ ^]^ On the other hand, metallomesogens with rigid disk‐shaped cores generally form columnar mesophases. For example, metallomesogens prepared by introducing metal ions into the central region of rigid cyclic compounds such as porphyrins and phthalocyanines have been observed to form columnar mesophases.^[^ ^10^ ^]^ Columnar metal complexes consisting of a quasi‐disk‐like core containing trivalent transition metals such as chromium (Cr^3+^), ruthenium (Ru^3+^), rhodium (Rh^3+^), and iron (Fe^3+^) and multiple ligands have also been studied.^[^ ^11^ ^]^ This columnar structure is formed by stacking highly symmetric octahedral coordination structures, i.e., 6‐coordinate geometries. Since metal‐metal and ligand‐derived π‐π interactions between cores are universally present in metallomesogens with symmetrical molecular structures, disk‐shaped metallomesogens generally form a columnar mesophase due to the interaction between rigid core complexes.^[^ ^12^ ^]^ The formation of a bicontinuous cubic mesophase is promoted by shifting the stack of the disk‐shaped metallomesogens.^[^ ^13^ ^]^ On the other hand, there have been only a few reports on the formation of micellar cubic mesophases, that is, supramolecular spherical arrays composed of disk‐shaped metallomesogens, and the detailed internal structure of these structures is unclear.^[^ ^14^ ^]^ Micellar cubic mesophases in solvent‐free systems were first discovered for spherical supramolecular assemblies of carbohydrate‐based compounds.^[^ ^15^ ^]^ A series of detailed analyses of formation mechanisms based on molecular design have revealed the involvement of hydrogen bonds in the formation of micellar cubic mesophases.^[^ ^16^ ^]^ It was also reported that some molecules form a micellar cubic phase at room temperature due to the structural rules of the side chains of the molecules.^[^ ^6b^ ^]^ The periodically ordered structure of spherical motifs composed of disk‐shaped molecules is thought to result from the controlled intermolecular interactions that contribute to the formation of the columnar mesophase.^[^ ^6a^ ^]^ Designing metallomesogens with control of the spatial self‐assembly requires following structural rules, and discovering spherical packing phases in metallomesogen‐based materials remains challenging.

**Figure 1:**
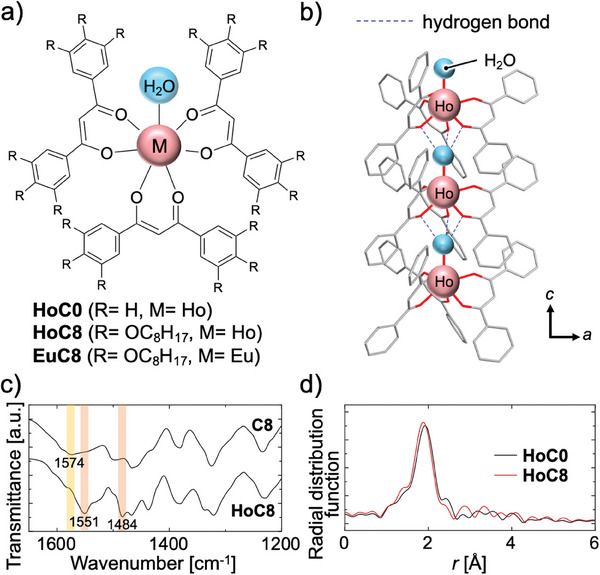
a) Structures of 7‐coordinate lanthanide complexes obtained by complexation with β‐diketone‐type ligands (**C0** and **C8**). b) Linearly aligned structure of the **HoC0** complex along the c‐axis in the crystal, based on **HoC0** single‐crystal data. The blue dotted lines indicate the predicted hydrogen bonds: the O…O interatomic distance is 2.957 Å, the O…O‐Ho angle is 143.77°, and hydrogen atoms are omitted. This figure is based on data from the Cambridge Crystallographic Data Center (No. 2270020). c) IR spectra of **C8** and **HoC8**. d) The radial distribution function of *k* ^3^‐weighted Ho L_3_‐edge EXAFS for **HoC0** and **HoC8**.

Lanthanide metals, which prefer high coordination numbers, show no marked stereochemical preference, and lanthanidomesogens form various assembled structures.^[^ ^17^ ^]^ In this context, we investigate the design of higher‐order structures based on lanthanide complexes. Holmium (Ho) is the lanthanide element with the highest magnetic moment, and magnetic materials such as single‐molecule magnets have been fabricated with Ho as the metallic species.^[^ ^18^ ^]^ We have developed Ho‐doped soft magnetic materials by incorporating carboxyl and β‐diketone groups with high complexation efficiency with lanthanides into the material design.^[^ ^19^ ^]^ Recently, we prepared a Ho complex (**HoC0**) consisting of Ho^3+^ and a β‐diketone‐type ligand, 1,3‐diphenyl‐1,3‐propanedione (**C0**) (**Figure** **1**a). While **HoC0** has been reported previously,^[^ ^20^ ^]^ the crystal structure of the newly synthesized sample was obtained for confirmation: single‐crystal X‐ray diffraction (SC‐XRD) measurements of **HoC0** showed that three **C0** ligands and one H_2_O molecule were coordinated around the central Ho atom.^[^ ^21^ ^]^ The H_2_O molecule formed hydrogen bonds with the carbonyl oxygen of the ligand of an adjacent Ho complex, thereby forming a linearly aligned structure of Ho complexes in the crystalline state (Figure 1b). Incorporating hydrogen bonds, where bonding and dissociation are reversible at room temperature, into the design of materials has proven useful in many fields.^[^ ^22^ ^]^ The unique nature of this self‐organization process of a 7‐coordinate lanthanide complex motivated us to further investigate the organization of Ho complexes into other assembled patterns.

Here, we present the discovery of a supramolecular spherical array based on a micellar cubic mesophase using lanthanide metallomesogens with 7‐coordinate geometry (Figure 1a). We assumed that appropriate control of the spatial extent of the peripheral side chains, combined with the reversible hydrogen bonding behavior at room temperature, promotes the self‐assembly of Ho complexes into the micellar mesophase. We thus prepared a **HoC8** complex composed of ligand **C8** with an 8‐carbon alkoxy chain as a side chain. Synchrotron X‐ray scattering experiments have revealed an unprecedented phenomenon: supramolecular spheres constructed by self‐assembly of three **HoC8** complexes directly form BCC‐type micellar cubic mesophases at room temperature without the formation of a columnar mesophase in which disk‐shaped molecules are generally formed. Using the molecular force field of Ho that we previously constructed,^[^ ^21^ ^]^ we verified the construction of a BCC structure consisting of **HoC8** complexes by molecular dynamics (MD) simulations. More importantly, this design concept was adapted to another lanthanide, europium (Eu), where **EuC8** directly formed BCC‐type micellar cubic mesophases at room temperature, similar to **HoC8**. Different lanthanides have different magnetic and luminescent properties,^[^ ^23^ ^]^ leading to the formation of arbitrary functional supramolecular spherical arrays. The results presented here highlight this innovative strategy to develop new supramolecular morphologies based on the conformational asymmetry of lanthanide complexes with high coordination geometry.

## 2 Results and Discussion

The Ho complex **HoC8** was prepared by combining the **C8** ligand possessing a flexible alkyl group on the side chain with holmium chloride hexahydrate (HoCl_3_·6H_2_O). The detailed experimental procedure is provided in the Supporting Information (Scheme S1 and Figure S1, Supporting Information). The coordination of the β‐diketone group to the Ho cation was investigated by infrared (IR) spectroscopy (Figure 1c). In the IR spectrum of **C8**, a strong absorption peak was observed at 1574 cm^−1^, which was attributed to the C═O stretching vibration of the β‐diketone moiety; in the spectrum of **HoC8**, this 1574 cm^−1^ peak redshifted to 1551 cm^−1^, indicating that the carbonyl group was coordinated to the Ho cation.^[^ ^24^ ^]^ In addition, a peak attributed to the C═C vibration was observed at 1484 cm^−1^ due to the deprotonation of the enol‐type hydroxyl group.^[^ ^19b^ ^]^ In the **EuC8** spectrum shown in Figure S2 (Supporting Information), as in that of **HoC8**, the peaks attributed to the C═O and C═C (enol isomer) stretching vibrations of the β‐diketone group of **C8** were redshifted after ligand coordination. Peaks attributed to C═C vibrations associated with the deprotonation of the enol hydroxyl group were also observed, indicating that the **C8** ligand was coordinated to Eu. The coordination state of Ho in **HoC8** was confirmed by X‐ray absorption fine structure (XAFS) spectroscopy. The extended X‐ray absorption fine structure (EXAFS) and X‐ray absorption near edge structure (XANES) spectra of the Ho L_3_‐edge of **HoC0** and **HoC8** shown in Figure S3a,b (Supporting Information) are almost identical, suggesting that the coordination state of Ho in both complexes was the same. The Fourier‐transformed (FT) *k* ^3^‐weighted Ho L_3_‐edge EXAFS functions, that is, the radial distribution functions of **HoC0** and **HoC8**, are shown in Figure 1d. For the strong peak at ≈2 Å attributed to Ho‐O coordination, curve‐fitting analysis was performed based on the crystal structure of Ho(III) oxide: the fitting result and graph are shown in Table S1 and Figure S3c,d (Supporting Information), respectively. There was no difference between the Debye‐Waller factors of **HoC8** and **HoC0**, indicating that the coordination environment for oxygen was nearly identical (Table S1, Supporting Information). Notably, the coordination numbers of Ho in **HoC0** and **HoC8** were equal. Thus, **HoC8** was a 7‐coordinate complex with three ligands and one H_2_O molecule coordinated to the central Ho, similar to **HoC0**.^[^ ^21^ ^]^

**Figure 2:**
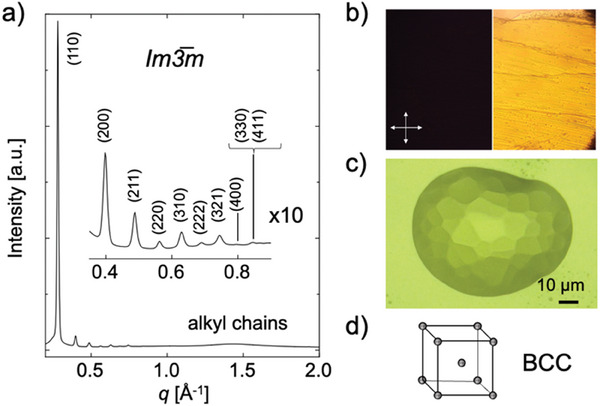
a) SAXS profile of **HoC8** at 25 °C. b) POM image (left) and optical microscopy image (right) of **HoC8** coated on a glass substrate at 25 °C. c) Optical micrograph at 25 °C of the equilibrium crystal habits of **HoC8** droplets prepared by slowly cooling (0.01 °C min^−1^) the isotropic liquid (200 °C). d) Schematic of a BCC structure.

Synchrotron small‐angle X‐ray scattering (SAXS) measurements were performed to clarify the detailed phase structures. As shown in **Figure** **2**a, the SAXS data for **HoC8** at room temperature (25 °C) showed a considerable number of well‐resolved reflections with *q*‐spacing ratios of $\sqrt{2}$, $\sqrt{4}$, $\sqrt{6}$, $\sqrt{8}$, $\sqrt{10}$, $\sqrt{12}$, $\sqrt{14}$, $\sqrt{16}$, and $\sqrt{18}$. By fitting the results to a cubic lattice, these peaks were indexed to the (110), (200), (211), (220), (310), (222), (321), (400), and (330/411) planes of a structure in the *Im*3̅*m* space group (Table S2, Supporting Information). There was a broad peak at ≈1.4 Å^−1^ (2*θ* = 13.2°, λ = 1.03 Å) attributed to the alkyl side chain of **HoC8**, indicating the mesomorphic nature of the sample.^[^ ^25^ ^]^ Whether the *Im3̅m* cubic mesophase was bicontinuous or micellar will be discussed later. The optical microscopy image of **HoC8** showed a hard texture (Figure 2b right). The dark‐field image obtained by polarized optical microscopy (POM) between crossed polarizers of the same field of view supported the presence of the cubic mesophase that was isotropic in three dimensions and showed no birefringence (Figure 2b left).^[^ ^26^ ^]^ Equilibrium crystal habits obtained by extremely slow cooling of cubic mesophase droplets from the isotropic phase are known to form polyhedral structures with crystallographic facets.^[^ ^27^ ^]^ Optical microscopy of the equilibrium crystal habits of the **HoC8** droplet revealed a faceted polyhedral structure, indicating that **HoC8** formed cubic mesophases at room temperature (Figure 2c). The SAXS profile for **EuC8** also showed that this material formed a cubic mesophase in the *Im3̅m* space group (Figure S4 and Table S3, Supporting Information). From the dimensions of the (110) reflections, the cubic lattice sizes of **HoC8** and **EuC8** at room temperature were estimated to be 31.34 and 30.87 Å, indicating a nearly constant size regardless of the size of the lanthanide elements. Cubic mesophases can be classified into two types: bicontinuous types, which are formed by a continuous arrangement of molecules, and discontinuous micellar types, which are formed by spherical assemblies.^[^ ^28^ ^]^ From the SAXS measurements, only the symmetry (space group) of the cubic mesophase was determined. Thus, to determine whether the cubic mesophase was bicontinuous or discontinuous, the number of **HoC8** complexes in the lattice (*Z* value) was calculated using the following Equation (1):

(1) $$Z\;=\;\frac{a^{3}\rho N_{A}}{M}$$

where *Z* is the number of molecules (complexes) in the lattice, *a* is the lattice constant, *ρ* is the density, *N*_A_ is Avogadro's number, and *M* is the molar mass. Using the density of **HoC8** (1.03 g cm^−3^) measured by means of a gas displacement pycnometer system with helium gas, the *Z* value was calculated to be 6.04. It is difficult for approximately six complexes to form a bicontinuous cubic mesophase, which requires the self‐assembly of many molecules,^[^ ^28^ ^]^ suggesting that **HoC8** forms a discontinuous micellar cubic mesophase. These results confirmed the formation of BCC‐type supramolecular spherical arrays in which spherical assemblies consisting of three **HoC8** complexes occupy two sites of the lattice (Figure 2d). The morphology of micellar cubic mesophases is known to strongly depend on the interfacial curvature of molecular aggregates: the smaller the interfacial curvature, the more bicontinuous cubic mesophases are formed, and the larger the interfacial curvature, the more discontinuous spherical mesophases tend to be formed.^[^ ^6^, ^15^, ^16^ ^]^ The three alkyl chains introduced into the side chain of each benzene ring of **C8** contribute to the increase of the interfacial curvature, which probably led to the formation of the micellar cubic mesophase consisting of **HoC8**. The *Z* value of **EuC8** was also ≈6, indicating that BCC‐type supramolecular spherical arrays formed spontaneously at room temperature regardless of the lanthanide type.

**Figure 3:**
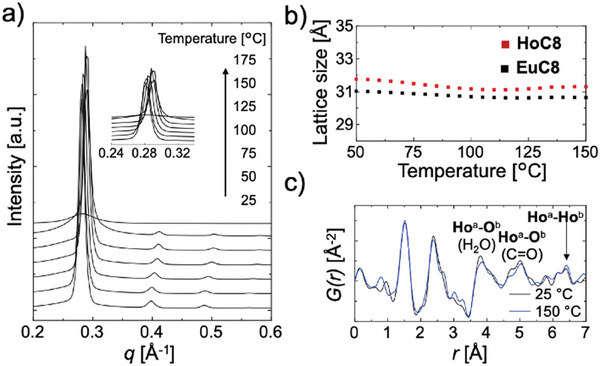
a) Temperature dependence of the SAXS profile of **HoC8**. b) Temperature dependence of the **HoC8** and **EuC8** lattice sizes. The lattice sizes were calculated from the peaks corresponding to the (110) reflections. c) PDF of **HoC8** X‐ray total scattering measurement data.

**Figure** **3**a shows the SAXS profiles of **HoC8** at various temperatures. All the profiles maintained the *Im3̅m* micellar cubic mesophase structure, although the peaks shifted slightly from 25 to 150 °C. At 175 °C, a change to an isotropic phase was observed. As shown in Figure S5 (Supporting Information), a peak near *q* = 1.4 Å^−1^ (*d* = 4.5 Å), which was attributed to the alkyl chain, was also observed at temperatures up to 150 °C, indicating that the mesomorphic properties were maintained up to this temperature range. Figure 3b shows the temperature dependence of the lattice size calculated from the peak corresponding to the (110) reflection at 50–150 °C. The lattice size of **HoC8** was 31.76 Å (50 °C), which decreased slightly to 31.30 Å (150 °C) upon heating. The change in the lattice size of **EuC8** showed a similar trend, with a slight decrease from 31.04 Å (50 °C) to 30.63 Å (150 °C) with increasing temperature. Compared to phase transitions, which result in significant changes in the ordered structure and mobility of materials, enthalpy changes due to changes in the lattice size are negligible. Thus, the lack of a distinct phase transition‐derived peak in the differential scanning calorimetry (DSC) curve for **HoC8** and **EuC8** also suggested that the peak shift in the SAXS profile was due solely to a change in lattice size (Figure S6, Supporting Information). The thermogravimetric (TG) curves of **HoC8** and **EuC8** showed that the decomposition temperatures were above 300 °C (Figure S7, Supporting Information). The fluidity of **HoC8** and **EuC8** was visually investigated using an optical microscope because no clear peaks were observed in the DSC curves for the prepared complexes, as described above. While **HoC8** and **EuC8** exhibited a hard texture at room temperature (25 °C), their fluidity increased with increasing temperature (Figure S8, Supporting Information); the isotropization temperatures of **HoC8** and **EuC8** were estimated to be ≈170 and 165 °C, respectively. Note that the dark field of view of the cubic mesophase was maintained in the POM images of **HoC8** and **EuC8** in all temperature regions (Figure S8, Supporting Information). While the lattice size varied slightly with temperature, both **HoC8** and **EuC8** were shown to form BCC‐type supramolecular spherical arrays over a wide temperature range, including room temperature.

To determine whether **HoC8** forms an ordered structure inside a supramolecular sphere, the short‐range ordered structure inside the material, i.e., the interatomic distance, was visualized by reduced pair distribution function (PDF) analysis based on synchrotron X‐ray total scattering measurements.^[^ ^29^ ^]^ Several distinct peaks were observed in the PDF of **HoC8** shown in Figure 3c. Referring to the interatomic distances in the SC‐XRD data for **HoC0**, the peaks below 3 Å were mainly correlated to atomic pairs within a single Ho complex. On the other hand, the distance between adjacent Ho complexes was ≈6.3 Å (Figure S9, Supporting Information). Therefore, the long‐range correlations longer than 6 Å observed in Figure 3c indicated that Ho complexes may be adjacent to each other. Heavier elements with larger atomic numbers have more significant scattering coefficients and correlations.^[^ ^30^ ^]^ Thus, the long‐range observed correlations were mainly related to Ho and correlated with the Ho–O(H_2_O) (3.99 Å), Ho–O(C═O) (5.00 Å), and Ho–Ho pairs (6.39 Å) between adjacent **HoC8** complexes. These data indicate that the three **HoC8** complexes in the supramolecular sphere are not arranged randomly, but the complexes form a linearly aligned structure. Notably, the peaks at 25 °C were also observed at 150 °C, albeit with a slight shift due to thermal motion, indicating that the 7‐coordinate structure of **HoC8** and the linearly aligned complexes it forms were maintained after heating because of the stability of the H_2_O molecules coordinated to the lanthanides.^[^ ^31^ ^]^ While the aligned complex structure inside the sphere center was maintained upon heating, the increased mobility of the alkyl side chains suggested that the supramolecular spheres converged to a more stable structure, and the lattice size was reduced (Figure 3b). The assembly mechanism of the 7‐coordinate **HoC8** was distinct from that of conventional symmetric transition metallomesogens. The Ho‐Ho distances between neighboring complexes were too great, presumably suppressing columnar assembly due to the lack of metal‐metal interactions.^[^ ^32^ ^]^ On the other hand, the presence of Ho–O(H_2_O) pair signals corresponding to hydrogen bonding intercomplexes suggests that the self‐assembly of **HoC8** was due to hydrogen bond through H_2_O molecules within the complex, quite similar to the internal structure of **HoC0** revealed by SC‐XRD measurements.

**Figure 4:**
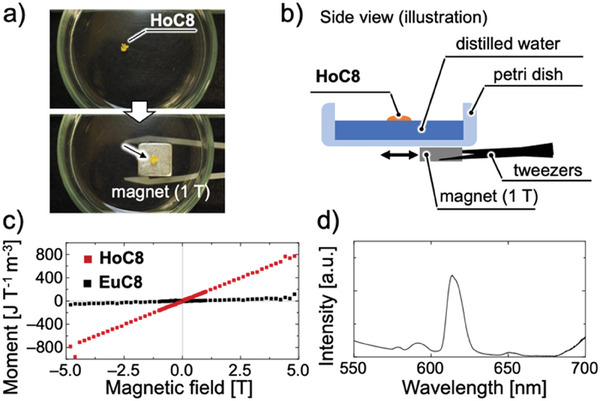
a) Photograph and b) schematic image of the magnetic responsive behavior of **HoC8**. See Movie S1 (Supporting Information) for the video. c) Magnetization plots of **HoC8** and **EuC8** in magnetic fields up to 5 T at 27 °C. d) The emission spectrum of **EuC8**. *λ*_ex_ = 370 nm.

Since Ho exhibits the highest magnetic moment among the lanthanide elements,^[^ ^23a^ ^]^ **HoC8** was floated on the water's surface in a petri dish to evaluate the sample's magnetic properties (**Figure** **4**a,b). Although the density of **HoC8** is 1.03 g cm^−3^, the sample floated on the water surface, probably due to the presence of tiny bubbles. When a neodymium magnet (1 T) approached from below the petri dish, the **HoC8** on the water surface moved with the movement of the magnet and showed excellent magnetic properties (Movie S1, Supporting Information). The mass magnetization was measured by superconducting quantum interference device (SQUID) measurements. Figure 4c shows the magnetization plots of **HoC8** and **EuC8** at 27 °C. Both passed through the origin at magnetic fields up to 5 T, indicating that both **HoC8** and **EuC8** are paramagnetic. The volume mass susceptibilities (*χ*) of **HoC8** and **EuC8** calculated from the slope of the graph were 1.61 × 10^2^ and 1.06 × 10 J T^−2^ m^−3^, respectively, indicating that the magnetic properties can be controlled by the lanthanide elements used. Since Curie's law applies to paramagnetic behaviors, Curie's constant was determined from the following Equation (2):^[^ ^33^ ^]^

(2) $$\chi \;=\;\frac{C}{T}$$

where *χ* is the volume magnetic susceptibility, *T* is the absolute temperature, and *C* is Curie's constant of the material. Using the calculated Curie's constant, the magnetic moment per complex was calculated from the following Langevin function (3):^[^ ^33^ ^]^

(3) $$\mu \;=\;\sqrt{\frac{\;3\mathrm{VC}k_{B}}{N}}$$

where *µ* is the magnetic moment, *V* is the volume of the unit lattice, *k*_B_ is Boltzmann's constant, and *N* is the number of complexes in a cubic lattice volume (*Z* value). The obtained magnetic properties of **HoC8** and **EuC8** are listed in **Table** **1**, along with each element's previously reported magnetic moment values. The experimental values of the magnetic moments (*µ*) of **HoC8** and **EuC8** were 10.9 *µ*_B_ and 2.8 *µ*_B_, respectively. These magnetic moments were calculated using the experimentally determined number of complexes in the unit lattice and the *Z* value. The obtained values are similar to the previously reported value of the effective paramagnetic moment of trivalent lanthanide ions,^[^ ^23a^ ^]^ supporting the formation of a supramolecular sphere from three complexes. These results also indicated that cubic metallomesogens were constructed using the prepared lanthanide complexes as building blocks. Since Eu is known to have luminescence properties, although its magnetic properties are weak, we measured the emission spectrum of **EuC8**. As shown in Figure 4d, a sharp peak was observed at a wavelength corresponding to the red color characteristic of Eu, indicating that the material inherited the properties of the element used.

**Table 1:** Magnetic properties of **HoC8** and **EuC8**.

Sample	*χ* [J T^−2^ m^−3^]	*C* [J K T^−2^ m^−3^]	*µ*/*µ_B_*	
			experimental	literature^[^ ^23a^ ^]^ ^a)^
**HoC8**	1.61 × 10^2^	4.84 × 10^4^	10.9	10.4–10.7
**EuC8**	1.06 × 10	3.17 × 10^3^	2.8	3.3–3.5

^a)^ Values of effective paramagnetic moment of trivalent lanthanide ions.

**Figure 5:**
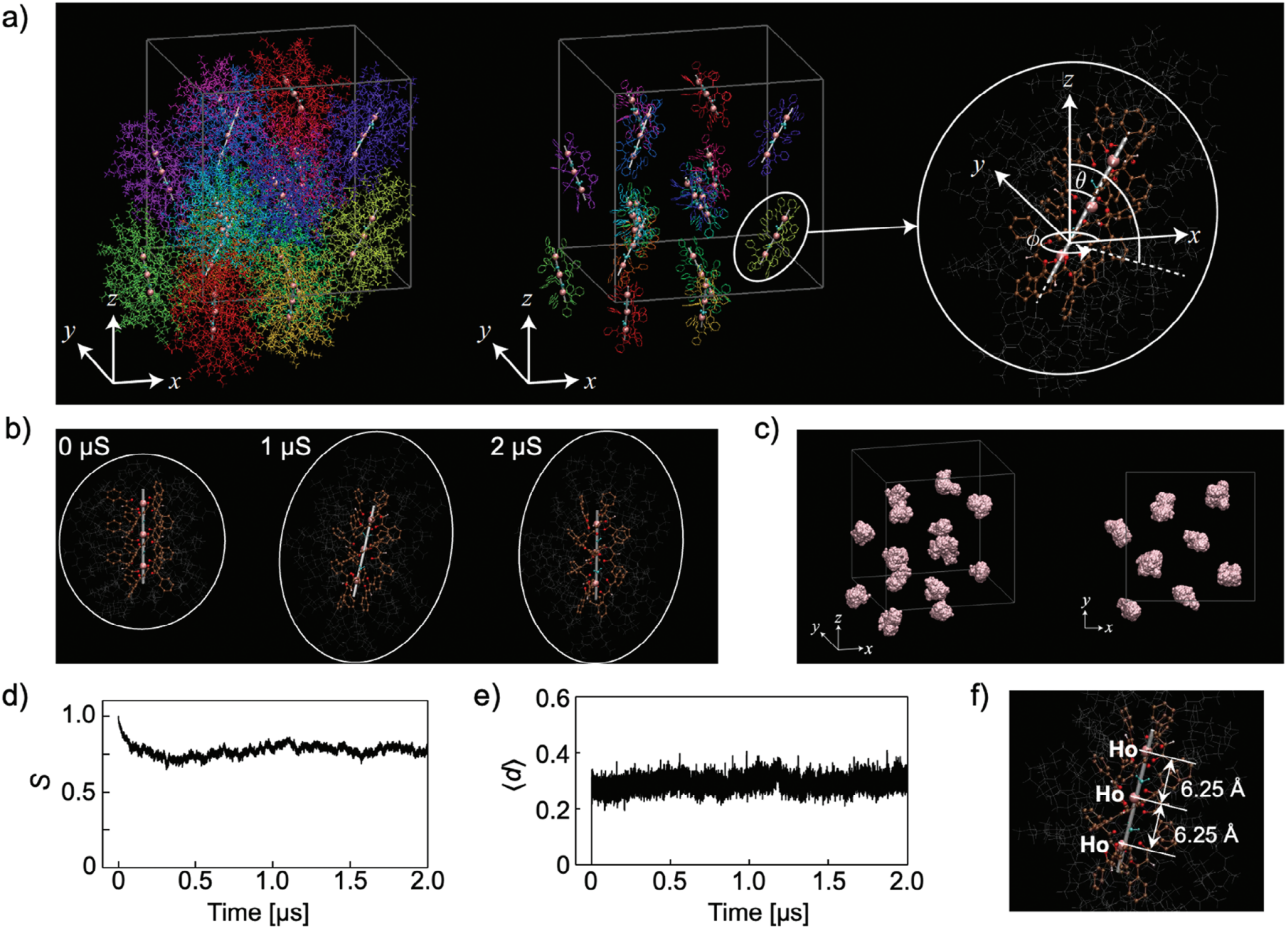
a) The snapshot at the final MD step is displayed with and without alkyl chains (left and middle images). Each assembly is colored with different colors to facilitate the orientation. An assembly is shown in the right image, where the pink, brown, and white spheres represent Ho, C, and H atoms, respectively. Gray lines indicate the alkyl chains. Cyan‐colored molecules correspond to water molecules. Dark gray lines denote the periodic boundaries of the unit cell. b) Time sequence of MD‐derived structures: the white enclosure represents the assembly spread. c) Centroid trajectory for each assembly: the pink sphere in the unit cell represents the centroid position at each time. d) The time‐dependent order parameter and e) the mean distance for three Ho and two O water molecules from the central axis of the assembly. f) Magnified image of the three **HoC8** complexes inside the assembly. The numbers in the figure indicate the nearest Ho–Ho distance.

We performed MD simulations to evaluate the validity of the BCC structure, which was experimentally shown to form by spontaneous assembly of supramolecular spheres composed of three **HoC8** complexes (Movie S2, Supporting Information). The atomic structure derived from the MD simulation at the final step was visualized using VMD software,^[^ ^34^ ^]^ as shown in **Figure** **5**a. The left and middle images in Figure 5a were drawn with different colors for each assembly. The middle image was drawn without alkyl chains to identify its orientation. The right image shows a single assembly with three **HoC8** complexes. The white straight lines in Figure 5a indicate the central axis through three Ho and two H_2_O molecules. The central lines for each assembly were determined from the coordination of Ho and O of H_2_O by solving the optimization problem described later. The time evolution of the structures from the initial structure is illustrated with a time sequence in Figures S10 and S11 (Supporting Information). An assembly including alkyl chains in the initial structure formed a spheroid centered around the central axis. Due to interactions between assemblies, the spheroid was transformed into an elongated shape with a major axis (Figure 5b). Visual inspection of the snapshots revealed that tilting and rotational motions occurred over time. The orientation of each assembly was defined within a spherical coordinate system, as shown in the right image of Figure 5a. The polar and azimuthal angles (*θ* (0° ≤ *θ* ≤ 180°) and *ϕ* (0° ≤ *ϕ* ≤ 360°), respectively) were calculated from the central axis of each assembly. The perpendicular distance from the central axis to three Ho atoms and two O atoms of H_2_O molecules was estimated to determine the central axis vector. The centroid position, ***o*** *_i_*, and the central axis vector were calculated by the following expression (4) involving rotational and translational operations:

(4) $$\underset{\vartheta ,\;\phi ,\;O_{i}}{\arg\min}\left\{\sum_{i}dist\left[o_{i},\;R_{x}\left(\vartheta \right)\;\cdot \;R_{z}\left(\phi \right)\cdot \left(r_{i}-\;o_{i}\right)\right]\right\}$$

where *dist*[***p***, ***q***] is the distance function used to calculate the distance between ***p*** and ***q*** in the *xy*‐plane. ***r*** *_i_* and ***o*** *_i_* are the coordinates of the *i*‐th atoms (three Ho atoms and two O atoms of H_2_O molecules) and the centroid of the central axis vector. *R_x_* (*θ*) and *R_z_* (*ϕ*) are the rotation matrices around the *x*‐ and z‐axes. Consequently, *R_x_* (*θ*) · *R_z_* (*ϕ*) · (***r*** *_i_* − ***o*** *_i_*) denotes the coordinates of the *i*‐th atoms after translational and rotational operations. All *i*‐th atoms after these operations were coordinated around the *z*‐axis when the argmin optimization problem in Equation (4) was solved. The central axis vector represented by *θ*, *ϕ*, and ***o*** *_i_* was determined at this point. The centroid displacements for each assembly were visualized by drawing the trajectory of ***o*** *_i_* (Figure 5c). The trajectories of ***o*** *_i_* encircled the lattice points in the BCC structure; thus, the centroid of the assemblies was positioned within the BCC structure. The time‐dependent *θ* of each assembly is displayed in Figure S13 (Supporting Information), where *m* in the panel corresponds to the label for each assembly shown in Figure S12 (Supporting Information). As the MD simulation progressed, all assemblies tilted from *θ* = 0 in the initial alignment to a range of 10 to 70°. Tilt angles exceeding 90°, which correspond to a complete inversion of an assembly with the head and tail swapped, were not observed.

The mean and standard error of *θ* for all assemblies after 0.6 µs were 28.9° ± 12.3°. The time‐dependent order parameter, denoted as *S*, was also calculated from *θ*(*t*) from Equation (5):

(5) $$S(t)\;=\;\frac{1}{2}\left(3\mathrm{co}s^{2}\theta (t)-\;1\right)$$

where ⟨ ⟩ represents the ensemble average for all assemblies (Figure 5d). The time‐dependent mean distance, denoted as ⟨*d*⟩ for the central axis, was also computed to assess the degree to which the three complexes and two H_2_O molecules aligned along a straight line (Figure 5e). *S* and ⟨*d*⟩ in the initial structure were 0, indicating that the central axes of all assemblies were perfectly aligned with the *z*‐axis. Although slight disordering with ⟨*d*⟩ ≈ 0.3 was observed, the three arrays of **HoC8** complexes along the central axis were maintained throughout the MD simulation. The distribution of *ϕ* for each assembly labeled as *m* in Figure S12 (Supporting Information) was visualized using a polar plot in Figure S14 (Supporting Information). The radial distance in Figure S14 (Supporting Information) indicates the distance between the head‐Ho and the tail‐Ho. The mean and standard error of this distance for all assemblies were 12.5 ± 0.3 Å, corresponding to a nearest Ho‐Ho distance of 6.25 Å (Figure 5f). From these results, the peak at ≈6.39 Å in Figure 3c was assigned to the distance between the nearest **HoC8** complexes. The small standard error of the head‐Ho‐to‐tail‐Ho length implies tight binding of three **HoC8** complexes along the central axis in the assembly. Some assemblies (*m* = 1,3 and *m* = 4) exhibited a *ϕ* distribution ranging from 0° to 360°, and other assemblies had a *ϕ* ranging from 45° to 180° centered in a specific direction. These observations indicated both rotational and librational motion around the *z*‐axis, meaning that the motion of the central axis was precessional.^[^ ^35^ ^]^ Such precessional motion is expected when an assembly is represented as a pseudospherical shape. As a result, the diffraction pattern of the crystal composed of **HoC8** complexes indicated the BCC structure.

**Figure 6:**
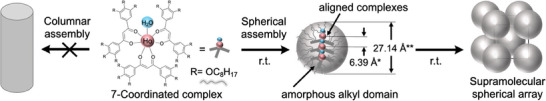
Schematic diagram of the mechanism of direct formation of a BCC‐type supramolecular spherical array at room temperature. *Measured by PDF analysis of X‐ray total scattering measurements. **Assuming that the supramolecular spheres are densely packed, we defined the diameter of the spheres as 1/2 the length of the diagonal of the lattice obtained from SAXS measurements.

A proposed mechanism for forming **HoC8**‐based supramolecular spherical arrays is summarized in **Figure** **6**. **HoC8** directly forms spherical assemblies consisting of three complexes. Inside the supramolecular sphere is the array structure of Ho complexes, surrounded by amorphous alkyl domains. These supramolecular spheres spontaneously align in an energetically stable orientation to form the BCC structure. **HoC8** does not form columnar assemblies, which disk‐shaped molecules tend to form, due to the unique 7‐coordinate geometry of the lanthanide complex. The asymmetry of the complexes and the presence of H_2_O molecules coordinated to Ho promote the formation of the aligned structure of the complexes driven by hydrogen bonds. The formation of a single supramolecular sphere with three **HoC8** complexes may be due to appropriate control of the spatial extent of the amorphous alkyl domains of the complex side chains. Nevertheless, the detailed formation mechanism of spherical assemblies still needs to be clarified. To elucidate the mechanism, it is necessary to comprehensively analyze the effects of side chains on the formation of higher‐order structures, and this research is currently underway. Importantly, all micellar cubic mesophase formation self‐assembly processes proceeded spontaneously at room temperature, indicating that complex structure formation was achieved via a low‐energy process.

## 3 Conclusion

In conclusion, experiments and MD simulations have shown that supramolecular spherical arrays composed of three 7‐coordinate lanthanide complexes form spontaneously at room temperature and create micellar mesophases directly without forming the columnar mesophases that conventional metal complexes tend to form. While there are limited examples of the preparation of *Im3̅m* micellar cubic mesophases,^[^ ^36^ ^]^ especially those obtained with metallomesogens as building blocks,^[^ ^37^ ^]^ we experimentally and computationally demonstrated the formation of *Im3̅m* micellar cubic mesophases based on lanthanide metallomesogens and the existence of ordered structures of metal complexes formed inside them. The supramolecular spherical arrays prepared with Ho possessed a high magnetic moment, as the central metal exhibited excellent magnetic properties that responded quickly to the movement of a bulk magnet. On the other hand, when Eu with a weak magnetic moment was used, the resulting supramolecular spherical arrays showed inferior magnetic properties, but red emission was observed. Since the magnetic and luminescence properties varied depending on the type of lanthanide element, it was possible to add any desired functionality by selecting the appropriate central metal. The newly developed method using 7‐coordinate lanthanide complexes as building blocks is expected to lead to the rational development of micellar mesophases other than the BCC type.

## Conflict of Interest

The authors declare no conflict of interest.

## Supporting information

Supporting Information

Supplemental Movie 1

Supplemental Movie 2

## Data Availability

The data that support the findings of this study are available on request from the corresponding author. The data are not publicly available due to privacy or ethical restrictions.
