# Supplementary material for: Magnetic Supramolecular Spherical Arrays: Direct Formation of Micellar Cubic Mesophase by Lanthanide Metallomesogens with 7‐Coordination Geometry
Source: Adv Sci (Weinh). 2024 Mar 13;11(20):2309226. doi: 10.1002/advs.202309226 (PMC11132039; doi:10.1002/advs.202309226)
Supplement: Supplementary file 1 — Supporting Information [file ADVS-11-2309226-s002.pdf]

ADVANCED SCIENCE
Open Access

Supporting Information

for *Adv. Sci.*, DOI 10.1002/advs.202309226

Magnetic Supramolecular Spherical Arrays: Direct Formation of Micellar Cubic Mesophase by Lanthanide Metallomesogens with 7-Coordination Geometry

Nao Komiyama, Takahiro Ohkubo, Yoshiki Maeda, Yuya Saeki, Nobuyuki Ichikuni, Hyuma Masu, Hirofumi Kanoh, Koji Ohara, Ryunosuke Takahashi, Hiroki Wadati, Hideaki Takagi, Yohei Miwa, Shoichi Kutsumizu, Keiki Kishikawa and Michinari Kohri**

Supporting Information

Magnetic Supramolecular Spherical Arrays: Direct Formation of Micellar Cubic Mesophase by Lanthanide Metallomesogens with 7-Coordination Geometry

Nao Komiyama, Takahiro Ohkubo, Yoshiki Maeda, Yuya Saeki, Nobuyuki Ichikuni, Hyuma Masu, Hirofumi Kanoh, Koji Ohara, Ryunosuke Takahashi, Hiroki Wadati, Hideaki Takagi, Yohei Miwa, Shoichi Kutsumizu, Keiki Kishikawa, and Michinari Kohri**

Experimental Section

Materials. Ethylene glycol dimethyl ether (DME), sodium hydride (NaH, dispersion in paraffin liquid), acetone, ethyl acetate, chloroform, *n*-hexane, holmium (III) chloride hexahydrate ($HoCl_3 \cdot 6H_2O$), and europium (III) chloride hexahydrate ($EuCl_3 \cdot 6H_2O$) were purchased from Kanto Chemical Co., Inc. (Tokyo, Japan). Ethanol, hydrochloric acid (HCl), and sodium ethoxide were obtained from FUJIFILM Wako Pure Chemical Industries, Ltd. (Osaka, Japan). 1,3-Diphenyl-1,3-propanedione was purchased from Tokyo Chemical Industry Co., Ltd. (Tokyo, Japan). Deionized water with a resistance of 18.2 MΩ·cm was obtained by passing through a Millipore Simplicity UV system. All other chemicals and solvents were of reagent grade and were used as received. Compounds **1**–**3** were synthesized according to the method described in the literature.[1–3]

Measurements. ^{1}H and ^{13}C nuclear magnetic resonance (NMR) spectra were measured at 400 MHz using a Bruker AVANCE III NMR spectrometer. Mass spectra were measured on an Exactive Thermo Fisher Scientific mass spectrometer. Fourier transform infrared (FT-IR) spectra were recorded on a JASCO FT/IR-4700 and Perkin Elmer Spectrum Two instruments. X-ray absorption fine structure (XAFS) spectra were measured in a transmission mode at BL15

of the SAGA Light Source, Kyushu Synchrotron Radiation Research Center. Small-angle X-ray scattering (SAXS) measurements were performed at the beamline BL10C at the Photon Factory (PF), the High Energy Accelerator Research Organization. X-ray total scattering measurements for pair distribution function (PDF) analysis were performed at the beamline BL04B2 of SPring8. Polarized optical microscopy (POM) was performed on a Nikon ECLIPSE E400 POL microscope equipped with an INSTEC HCS400 hot-cool stage, crossed polarizers, and analyzers. Magnetic properties were measured using a Quantum Design MPMS XL-5 superconducting quantum interference device (SQUID) magnetometer. Sample density was measured using a Shimadzu ACCUPYC II gas displacement pycnometer system with helium gas. The thermophysical properties of the sample were measured with a HITACHI DSC7020 differential scanning calorimetry (DSC) and a HITACHI NEXTA-STA thermogravimetric (TG) analyzer. Optical microscope images were obtained using a Keyence VHX-500F digital microscope. Elemental analytical measurements were performed using an Exeter Analytical CE-440F elemental analyzer.

Synthesis of 1,3-bis(3',4',5'-trioctyloxyphenyl)-1,3-propanedione (C8). The detailed synthetic scheme is shown in Scheme S1. Compound **3** (2.72 g, 5.38 mmol) dissolved in DME (20 mL) was added using a dropping funnel to a 200 mL quart flask containing DME (30 mL) and NaH (2.02 g, 50.4 mmol) and stirred for 30 min. Compound **1** (4.77 g, 9.16 mmol) dissolved in DME (20 mL) was then added to the mixture using a dropping funnel and stirred at 80 °C for 18 h under reflux conditions. The reaction mixture was slowly added to H_2O (300 mL) in an ice bath, and the excess NaH was treated with HCl aq. The sample was collected by suction filtration and washed with distilled water to obtain a yellow solid. The sample was recrystallized in ethyl acetate/hexane = 1/5 solution and then purified by silica gel chromatography using

chloroform as a solvent to afford **C8** (1.45 g, 1.46 mmol) in 27 % yield as yellow viscous material. ^{1}H NMR (400 MHz, $CDCl_3$, *δ*): 0.88 (*t*, *J* = 6.8 Hz, 18H), 1.22–1.55 (*m*, 60H), 1.80 (*m*, *J* = 7.3 Hz, 12H), 3.95–4.10 (*m*, 12H), 4.51 (*s*, 1H), 6.63 (*s*, 1H), 7.17 (*s*, 4H); ^{13}C NMR (100 MHz, $CDCl_3$, *δ*): 14.2, 22.7, 26.1, 29.4, 30.4, 31.9, 69.5, 73.7, 92.3, 106.2, 130.5, 142.4, 153.2, 185.3; ESI(+)-MS calcd. for $C_{63}H_{108}O_8Na$ $[M+Na]^+$: 1015.7936; found: 1015.7966.

Preparation of tris[1,3-bis(3',4',5'-trioctyloxyphenyl)-1,3-propanedionato]-aqua-holmium (HoC8). Sodium ethoxide (17.0 mg, 0.25 mmol, 1 mL ethanol solution) was added dropwise to an ethanol solution (20 mL) in which **C8** (0.24 g, 0.24 mmol) was dissolved, and then the mixture was refluxed for 30 min. A solution of $HoCl_3·6H_2O$ (30.4 mg, 0.08 mmol) in 1 mL of ethanol was added to the reaction mixture and refluxed for an additional 30 min. After cooling the sample to room temperature, the solvent was removed by decantation to give a viscous orange oil. The obtained oily sample was dissolved in hot acetone and allowed to stand at 15 °C. The solvent was removed by decantation to give **HoC8** as a viscous yellow solid (0.15 g). Anal. calcd. for $C_{189}H_{323}O_{25}Ho$: C 71.83, H 10.30; found: C 71.76, H 10.31. **EuC8** was prepared by the same procedure as **HoC8**, with $EuCl_3·6H_2O$ instead of $HoCl_3·6H_2O$. Anal. calcd. for $C_{189}H_{323}O_{25}Eu$: C 72.12, H 10.34: found: C 71.75, H 10.34.

Structural modeling. The crystalline **HoC8** structure was modeled using all-atomic molecular dynamics (MD) simulations to investigate the alignment of the **HoC8** complex. The second-generation force-field functions,[4,5] which can accurately reproduce the crystalline structure of organic compounds, were employed for the MD simulations. The force-field parameters were obtained from the original COMPASS dataset[4] and our own developed parameters for **HoC0** crystal.[6,7] These force-field parameters have been validated to accurately predict the crystalline

lattice constant, Ho local structure, and vibrational properties obtained from both experimental observations and first-principles calculations of **HoC0** crystal.

The initial structure for the MD simulations was constructed based on experimentally obtained results. The lattice shape was a body-centered cubic (BCC), containing six **HoC8** complexes within the unit cell. To maintain the translational symmetry of BCC, these six **HoC8** complexes were arranged into two assemblies; thus, three aggregated **HoC8** complexes as an assembly occupy the lattice points (0,0,0) and (1/2, 1/2, 1/2).

An assembly consisting of three **HoC8** complexes was constructed using the crystalline **HoC0** structure as a template. Three **HoC0** complexes aligned along the three-fold rotational axis were built from the **HoC0** crystalline structure.[6,7] This ensured that the distance between the nearest complex was equal to the lattice constant c of **HoC0** (6.2589 Å). Subsequently, alkyl chain groups were attached to the ligands in **HoC0** to be the **HoC8** complex.

The assembly containing three **HoC8** complexes (1611 atoms in total) was equilibrated as an isolated system to maintain the **HoC0** crystalline arrangement. All equilibration runs for the isolated assembly were conducted while keeping fixed Ho positions. First, structural optimization was performed to satisfy the energy and maximum force tolerances of 10^{-4} kcal/mol and 10^{-6} kcal/mol/Å, respectively. Next, an equilibrium MD run was performed at 300 K with variable timestep ranging from short (0.001 fs) to standard (1.0 fs). Following energy equilibration at 300 K for 100 ps, the system was gradually cooled to 3 K over 100 ps. The assembly at the final step was employed as a component in constructing the initial **HoC8** structure with a BCC unit cell.

The unit cell of **HoC8** with 2 × 2 × 2 supercell with BCC lattice, which has sixteen assemblies with 25776 atoms in total, was created by occupying the lattice points with the assemblies. An equilibrated assembly made from the isolated system was placed intact without any rotational

operation; in other words, the three-fold axis of all assemblies was aligned in the *z*-direction. The constructed unit cell was first optimized to satisfy the tolerance of maximum force 1×10^{-8} kcal/mol/Å. Subsequently, an MD run was performed with 2.0 fs timestep under an isothermal-isobaric (NPT) ensemble using a Nos´e–Hoover thermostat[8,9] at 300 K and isotropic 1.0 atmospheres pressure for 700 million steps corresponding to 2.0 µs. At this stage, all atoms, including Ho, were not fixed. The intra-molecular C–H and O–H bonds were constrained using the SHAKE algorithm[10] with a tolerance of 0.0001 Å. The all-atomic coordination was sampled every 100 ps for subsequent analysis.

Pre- and post-treatment codes for building the initial structure and for analyzing the MD-derived atomic configurations were developed in this study. All MD simulation was conducted with the LAMMPS code.[11,12] The initial structure and force-field parameters for MD were provided in Supporting Information along with LAMMPS input files (dango3-C8.in and dango3-C8.data).

All MD trajectory data and analysis codes in this study were available upon request to the corresponding authors.

Supplementary Scheme

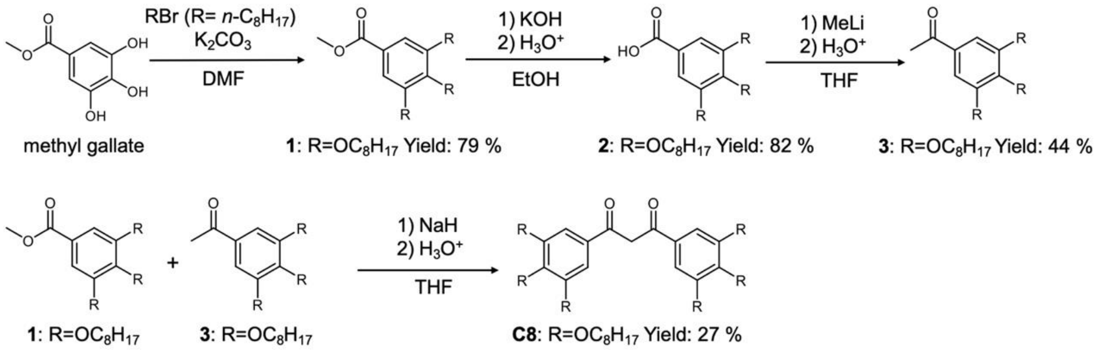

Scheme S1. Synthetic route of the β-diketone type ligand **C8**.

Supplementary Figures

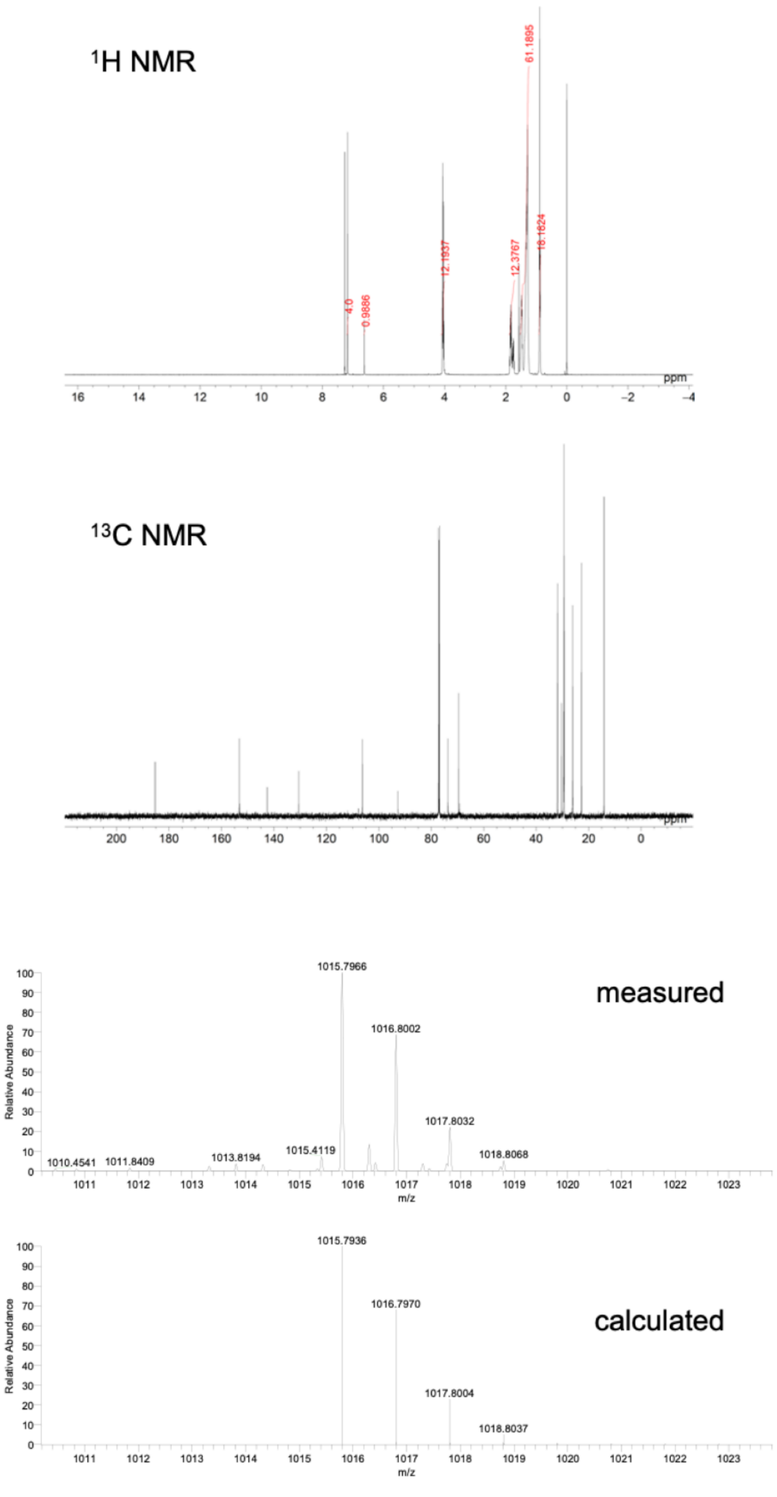

Figure S1. ^{1}H NMR, ^{13}C NMR, and ESI MS spectra for **C8**.

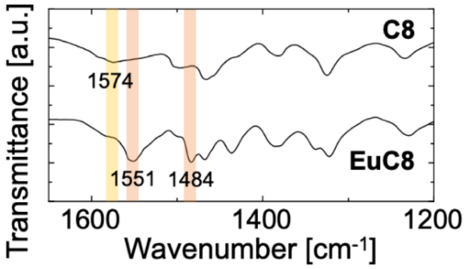

Figure S2. IR spectra for **C8** and **EuC8**.

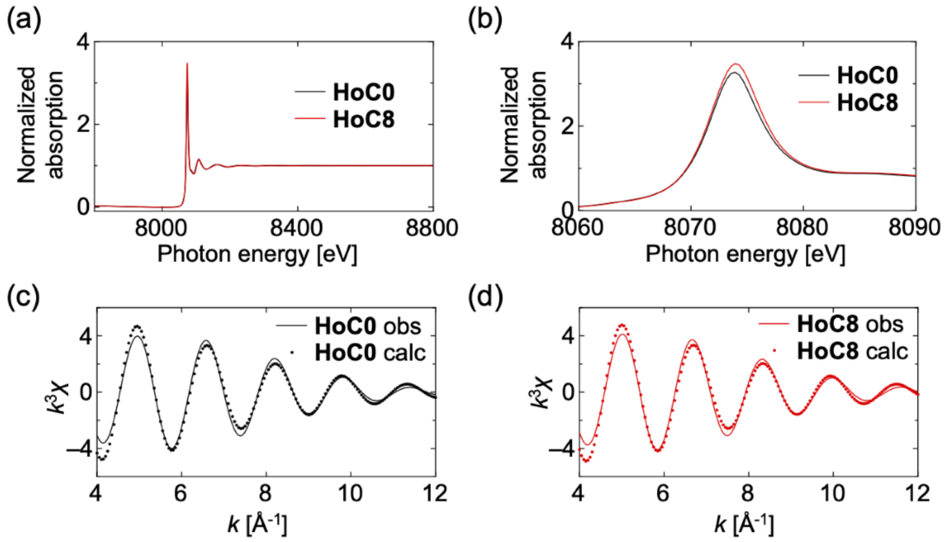

Figure S3. Ho L_3-edge (a) EXAFS and (b) XANES spectra for **HoC0** and **HoC8**. Curve-fitting results of Ho-O coordination for (c) **HoC0** and (d) **HoC8**.

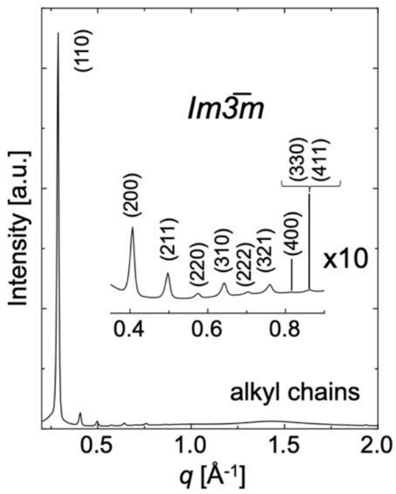

Figure S4. The SAXS profile for **EuC8** at 25 °C.

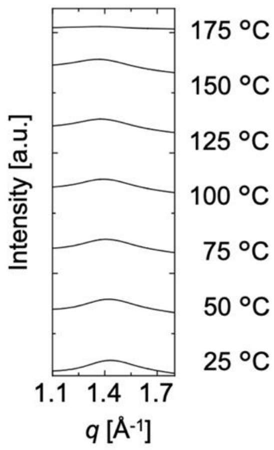

Figure S5. SAXS profiles for **HoC8** near q = 1.4 Å^{-1} (d = 4.5 Å) at various temperatures.

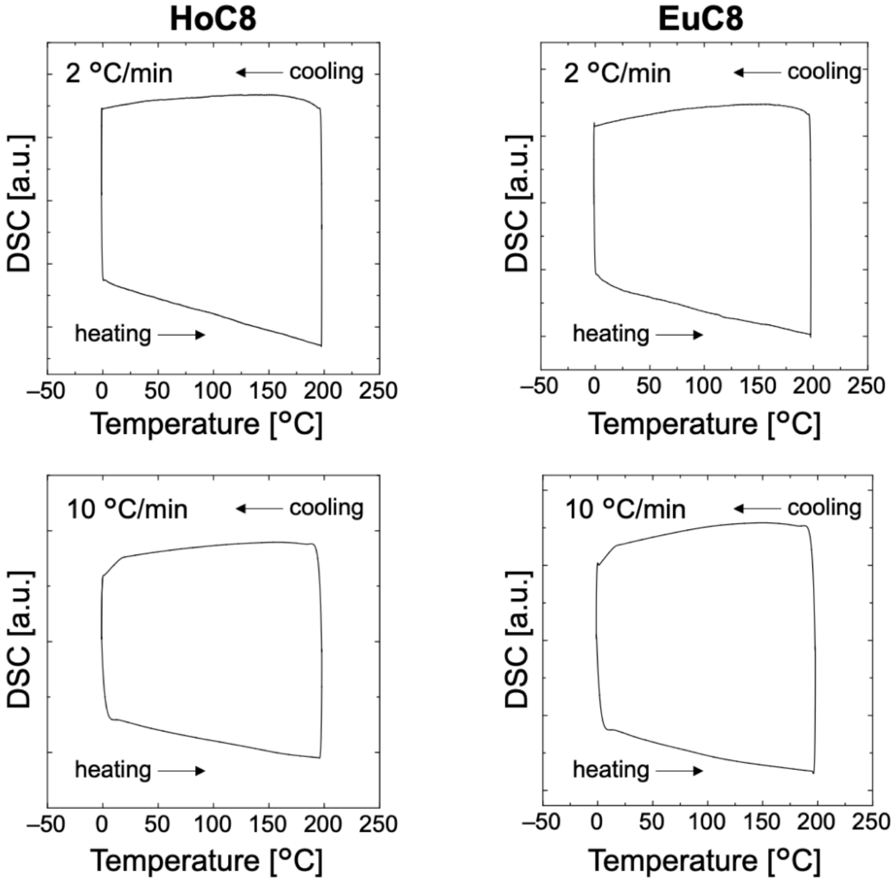

Figure S6. DSC profiles for **HoC8** and **EuC8**. Heating/cooling rates are 2 °C min^{-1} and 10 °C min^{-1}.

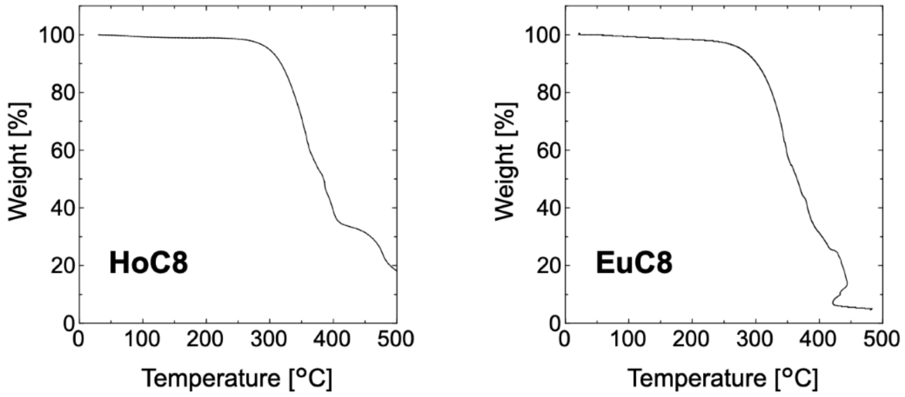

Figure S7. TG curves for **HoC8** and **EuC8** at a heating rate of 5 °C min^{-1}.

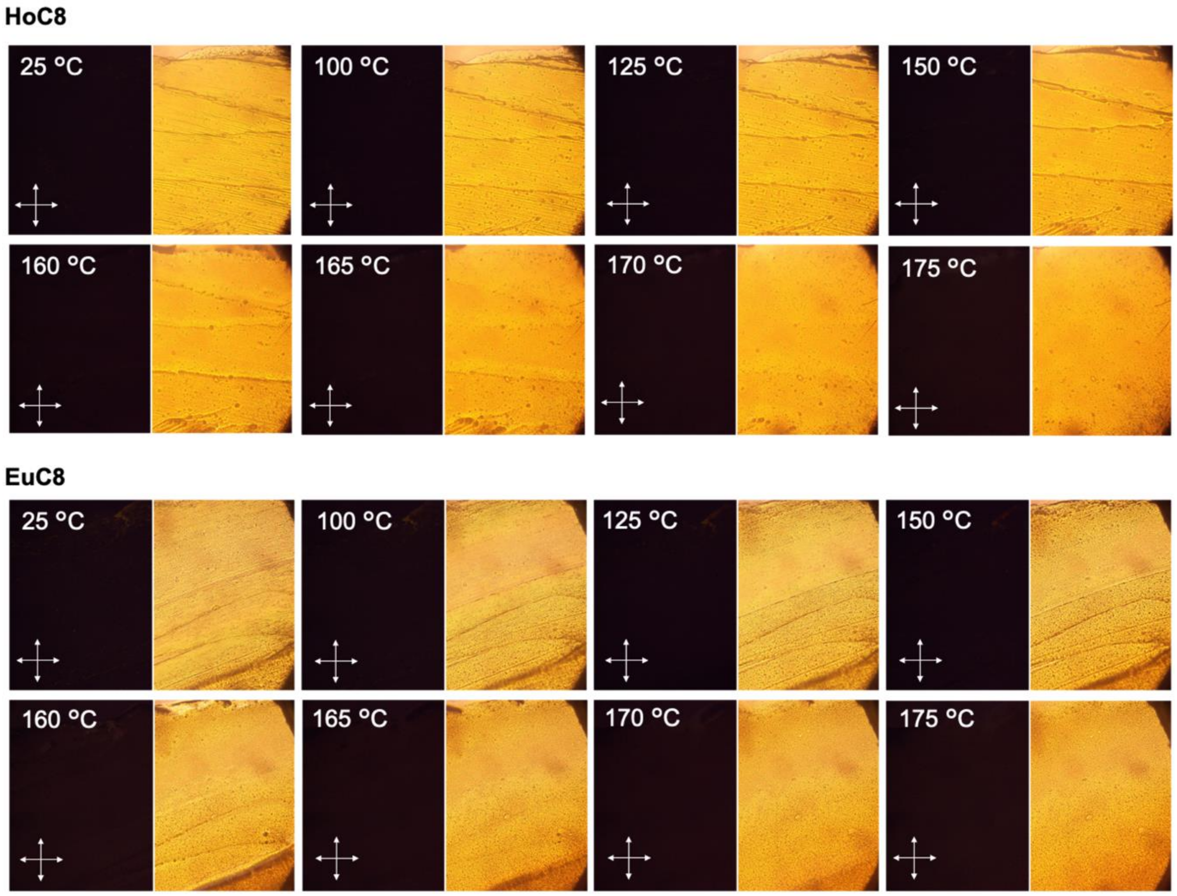

Figure S8. POM images (left) and optical microscope images (right) for **HoC8** and **EuC8** at different temperatures. The images on the left were obtained between crossed polarizers, while those on the right were taken without the polarizer. Observations were made while heating.

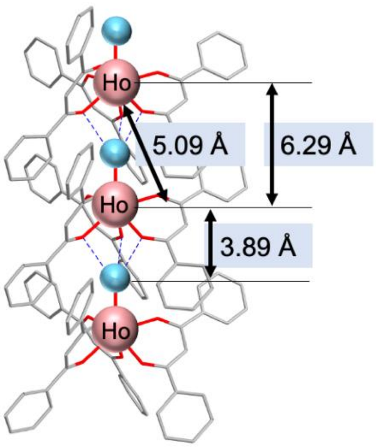

Figure S9. Distance between Ho-related element pairs calculated from SC-XRD measurements of **HoC0**. This figure is based on data from the Cambridge Crystallographic Data Centre (No. 2270020).

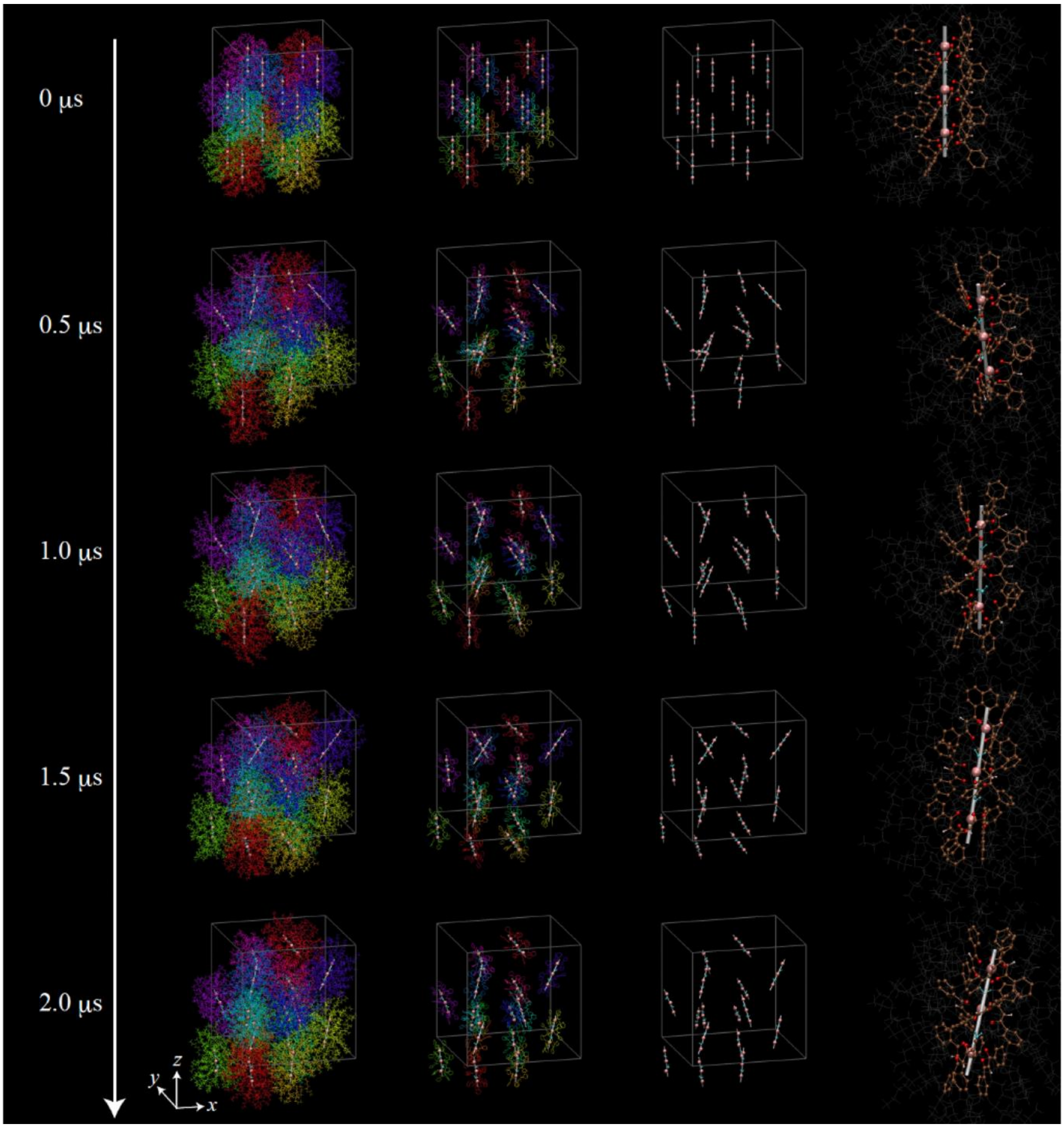

Figure S10. Time sequence of MD-derived structures. The left images show all atoms with different color for each assembly. The images in second and third columns indicate atomic structure without alkyl chains and only Ho and water molecules. A single assembly were drawn with the rightest columns, where the pink, brown, and white spheres represent Ho, C, and H atoms, respectively. Gray lines indicate the alkyl chains. All images are displayed with a 15° rotation in the xy- plane. The white straight lines on each assembly indicate the central axis vector.

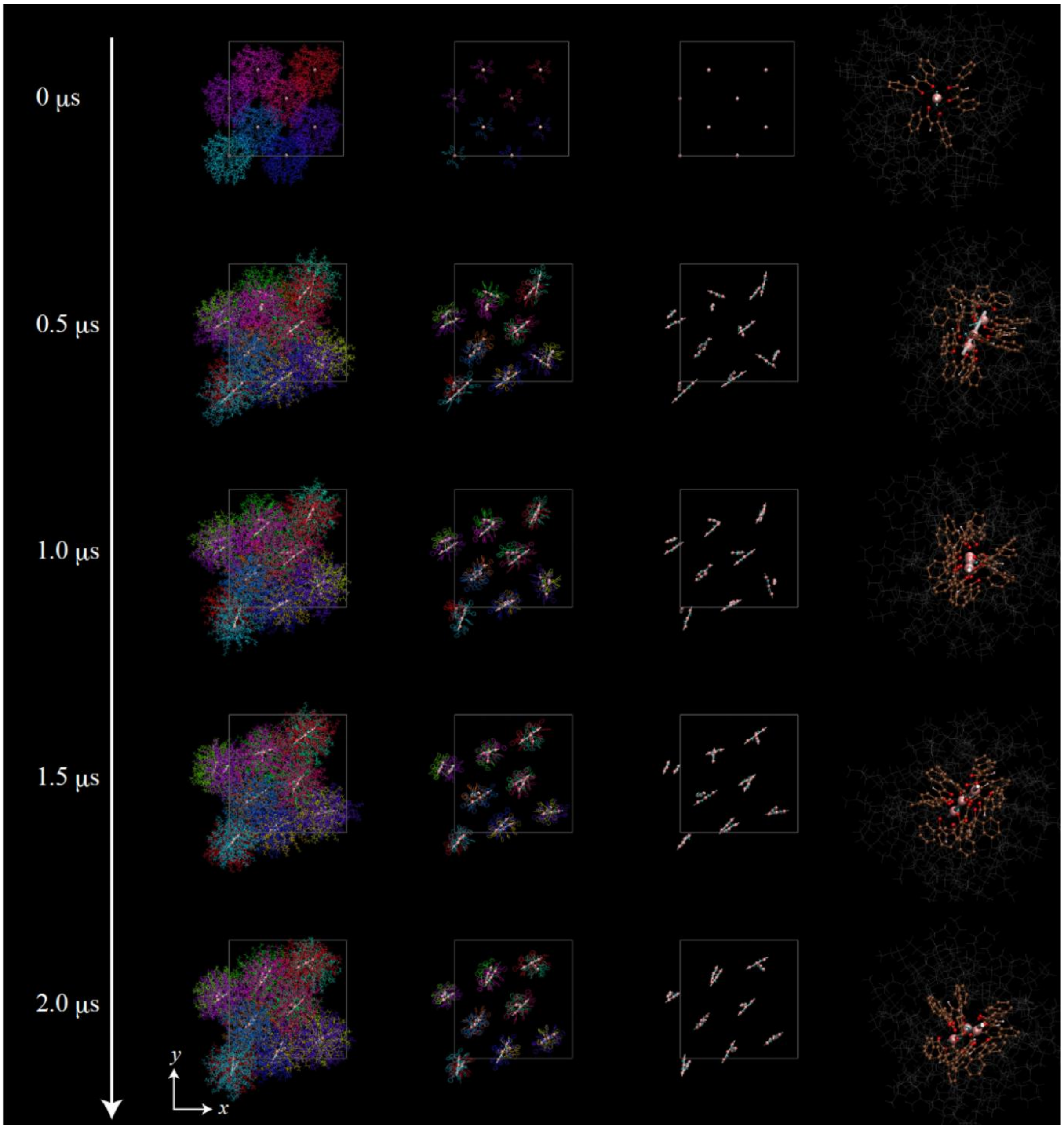

Figure S11. Time sequence of MD-derived structures. Figure S11 shows the time evolution of the same atomic structure as in Figure S10 in the *xy*-plane.

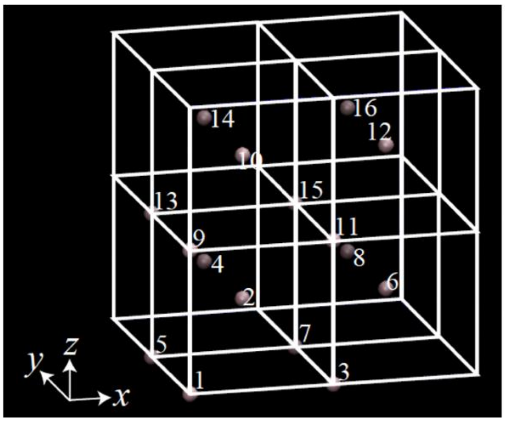

m	(x,y,z)	m	(x,y,z)
1	$(0,0,0)$	2	$(\frac{1}{4},\frac{1}{4},\frac{1}{4})$
3	$(\frac{1}{2},0,0)$	4	$(\frac{3}{4},\frac{1}{4},\frac{1}{4})$
5	$(0,\frac{1}{2},0)$	6	$(\frac{1}{4},\frac{3}{4},\frac{1}{4})$
7	$(\frac{1}{2},\frac{1}{2},0)$	8	$(\frac{3}{4},\frac{3}{4},\frac{1}{4})$
9	$(0,0,\frac{1}{2})$	10	$(\frac{1}{4},\frac{1}{4},\frac{3}{4})$
11	$(\frac{1}{2},0,\frac{1}{2})$	12	$(\frac{3}{4},\frac{1}{4},\frac{3}{4})$
13	$(0,\frac{1}{2},\frac{1}{2})$	14	$(\frac{1}{4},\frac{3}{4},\frac{3}{4})$
15	$(\frac{1}{2},\frac{1}{2},\frac{1}{2})$	16	$(\frac{3}{4},\frac{3}{4},\frac{3}{4})$

Figure S12. The figure on the left shows the centroid positions of assemblies in the unit cell. The numbers in the figure indicate the numbers assigned to the assemblies in the unit lattice. The table on the right shows the fractional coordinates (*x*,*y*,*z*) of the centroid of each assembly for the initial structure of the 16 assemblies. The number *m* is label to identify assembly in Figures S13 and S14.

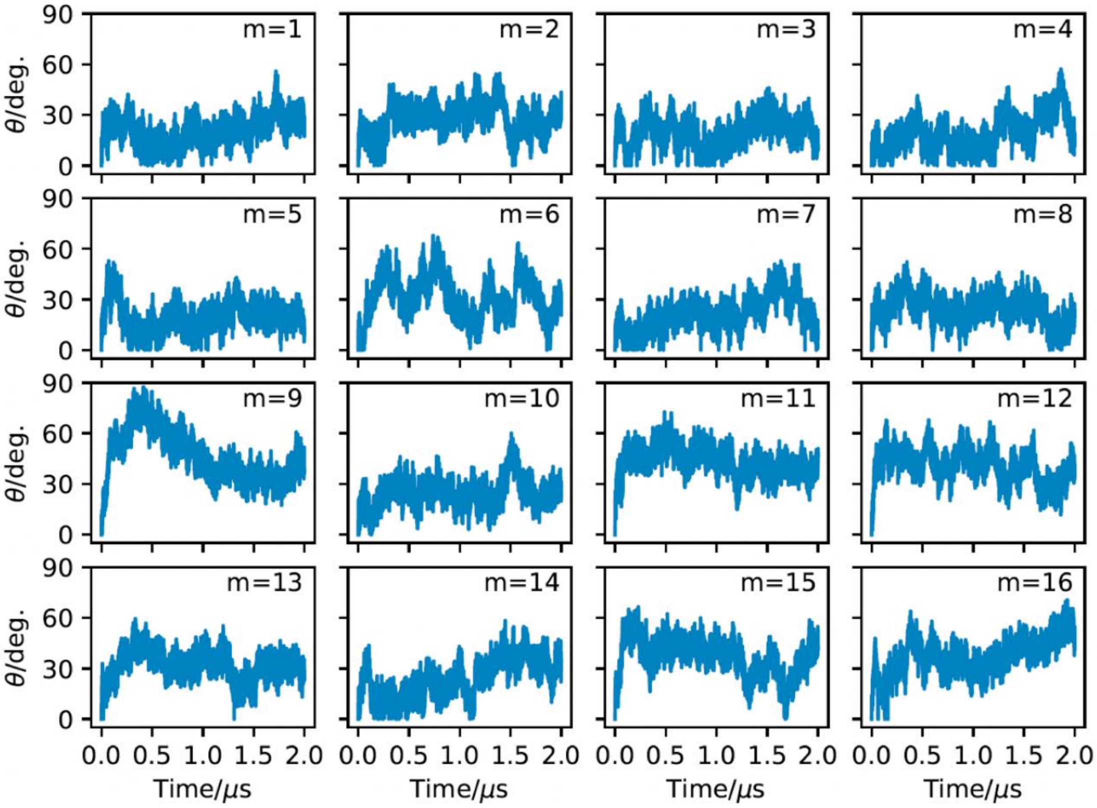

Figure S13. Time-dependent polar angles, θ, for each assembly. The number *m* in the panel is the identified label as indicated in Figure S12.

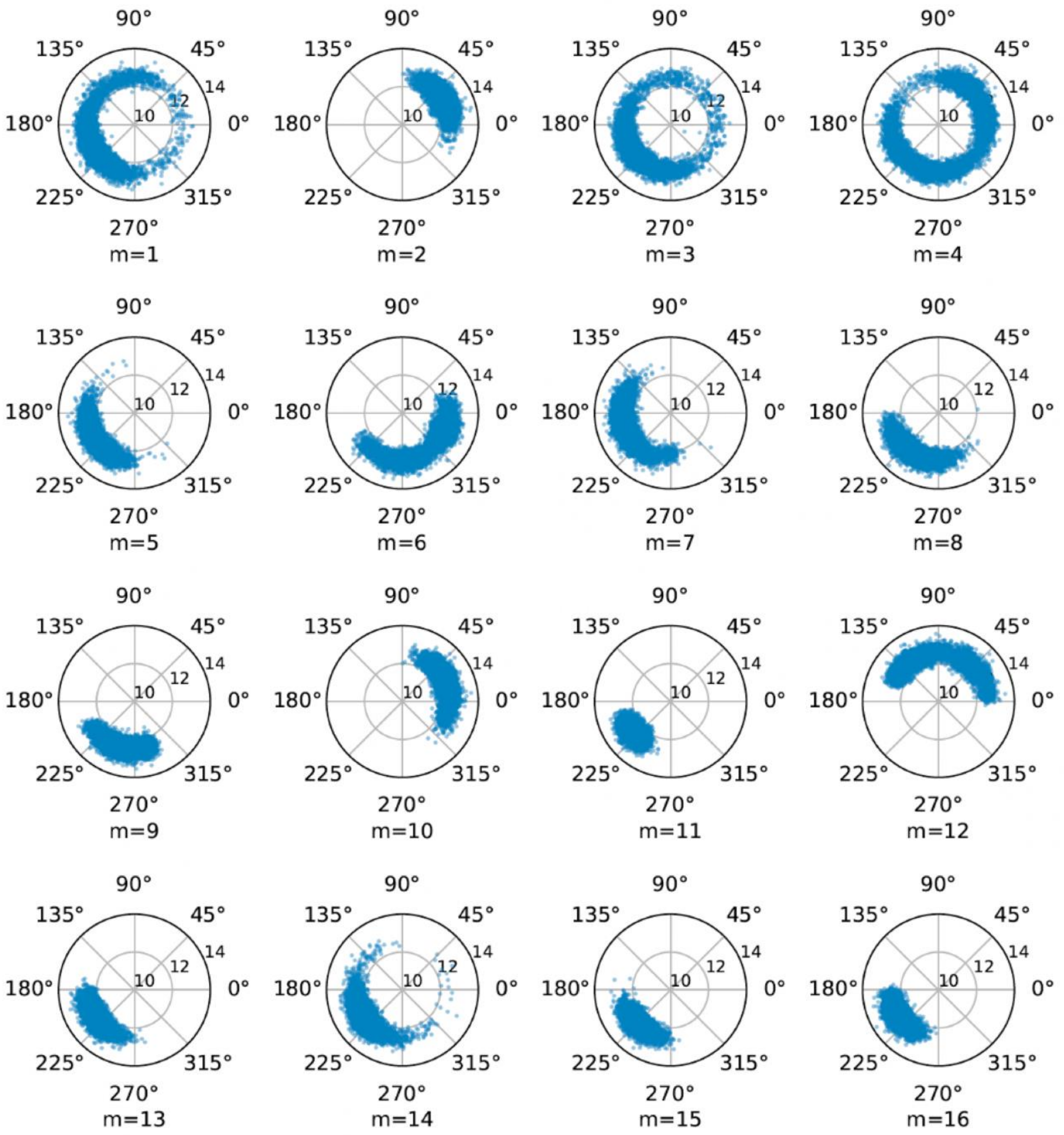

Figure S14. Azimuthal angles, ϕ, distributions for each assembly. The number m in the panel is the identified label, as indicated in Figure S12.

Supplementary Tables

Table S1. Curve fitting results for Ho-O coordination for **HoC0** and **HoC8**.

Sample	Filtering range [Å]	E_0 [eV]	CN[a]	R [Å][b]	dE [eV][c]	σ^2 [Å^2][d]
**HoC0**	1.55–2.33	8067.67	7.8±1.7	2.34±0.02	3.69±2.49	0.0105±0.0025
**HoC8**	1.51–2.32	8067.69	7.8±1.2	2.31±0.01	2.53±1.85	0.0104±0.0018

FT k-range: 3.0–12.0 Å^{-1}. Curve fitting analysis was carried out using the analysis program Athena and Artemis.[13] [a] Coordination number. [b] Coordination distance. [c] Differences between model and sample threshold energies. [d] Debye-Waller factor.

Table S2. SAXS data for **HoC8** at 25 °C.

hkl	$h^2+k^2+l^2$	q [Å^{-1}]	Spacing	
			$d_{obs.}$ [Å]	$d_{cal.}$ [Å]
110	2	0.283	22.16	22.16
200	4	0.398	15.80	15.67
211	6	0.488	12.87	12.80
220	8	0.564	11.15	11.08
310	10	0.633	9.93	9.91
222	12	0.691	9.09	9.05
321	14	0.747	8.41	8.38
400	16	0.801	7.85	7.84
330/411	18	0.848	7.41	7.39

Table S3. SAXS data for **EuC8** at 25 °C.

hkl	$h^2+k^2+l^2$	q [Å^{-1}]	Spacing	
			$d_{obs.}$ [Å]	$d_{cal.}$ [Å]
110	2	0.289	21.83	21.83
200	4	0.406	15.46	15.44
211	6	0.497	12.64	12.60
220	8	0.575	10.94	10.91
310	10	0.641	9.80	9.76
222	12	0.704	8.93	8.91
321	14	0.760	8.27	8.25
400	16	0.814	7.72	7.72
330/411	18	0.863	7.28	7.28

Supplementary Movies

Movie S1. Animation showing **HoC8** floating on the surface of the water and moving with the movement of the magnet.

Movie S2. MD simulation of a BCC structure formed by the spontaneous association of supramolecular spheres composed of three **HoC8** complexes.

References

[1] H. Zheng, C. K. Lai, and T. M. Swager, *Chem. Mater.* 1**995**, *7*, 2067–2077.

[2] B. Adelizzi, B. Adelizzi, P. Chidchob, N. Tanaka, B. A. G. Lamers, S. C. J. Meskers, S. Ogi, A. R. A. Palmans, S. Yamaguchi, and E. W. Meijer, *J. Am. Chem. Soc.* **2020**, *142*, 16681–16689.

[3] C. Liao, H. Chen, H. Hsu, A. Poloek, H. Yeh, Y. Chi, K. Wang, C. Lai, G. Lee, C. Shih, and P. Chou, *Chem. Eur. J.* **2011**, *17*, 546–556.

[4] H. Sun, *J. Phys. Chem. B* **1998**, *102*, 7338–7364.

[5] H. Heinz, T. J. Lin, R. Kishore Mishra, and F. S. Emami, *Langmuir* **2013**, *29*, 1754–1765.

[6] T. Ohkubo, N. Komiyama, H. Masu, K. Kishikawa, and M. Kohri, *Inorg. Chem.* **2023**, *62*, 11897–11909.

[7] A. Zalkin, D. H. Templeton, and D. G. Karraker, *Inorg. Chem.* **1969**, *8*, 2680–2684.

[8] S. A Nos´e, *J. Chem. Phys.* **1984**, *81*, 511–519.

[9] W. G. Hoover, *Phys. Rev. A.* **1985**, *31*, 1695–1697.

[10] J. P. Ryckaert, G. Ciccotti, and H. J. Berendsen, *J. Comput. Phys.* **1977**, *23*, 327–341.

[11] S. Plimpton, *J. Compt. Phys.* **1995**, *117*, 1–19.

[12] A. P. Thompson, H. M. Aktulga, R. Berger, D. S. Bolintineanu, W. M. Brown, P. S. Crozier, P. J. in ’t Veld, A. Kohlmeyer, S. G. Moore, T. D. Nguyen, R. Shan, M. J. Stevens, J. Tranchida, C. Trott, and S. J. Plimpton, *Comput. Phys. Commun.* **2022**, *271*, 108171.

[13] B. Ravel and M. Newville, *J. Synchrot. Radiat.*, **2005**, *12*, 537–541.
